# Weight underestimation for adults in Beijing and its association with chronic disease awareness and weight management

**DOI:** 10.1186/s12944-018-0873-7

**Published:** 2018-09-27

**Authors:** Kai Fang, Hang Li, Aijuan Ma, Jing Dong, Jin Xie, Ying Zhou, Kun Qi, Yingqi Wei, Gang Li, Jie Cao, Zhong Dong

**Affiliations:** 1 Beijing Municipal Center for Disease Prevention and Control, Beijing Research Center for Preventive Medicine, Beijing, China; 2 Department of Epidemiology, Fuwai Hospital, State Key Laboratory of Cardiovascular Disease, National Center for Cardiovascular Diseases, Chinese Academy of Medical Sciences and Peking Union Medical College, Beijing, China

**Keywords:** Overweight and obesity, Weight underestimation, Weight management

## Abstract

**Background:**

Obesity is known to be a risk factor to a variety of chronic diseases. Weight misperception has an impact on weight-loss attitude and behavior. We aimed to investigate factors associated with weight underestimation, and to assess the effect of hypertension, diabetes and dyslipidemia awareness on weight underestimation and weight management for overweight and obese adults.

**Methods:**

Data was obtained from the 2011 Beijing Non-communicable disease and risk factors Surveillance (BJNCDRS). A total of 19,932 participants with measures of weight and height were included in the analysis. Self-perception of weight was obtained by asking, “How do you describe your weight?”, and the question for weight management was “Are you taking any actions to control your body weight?”. Multiple logistic regression was used to investigate factors related to weight underestimation.

**Results:**

For the underweight, normal weight, and overweight/obese categories, more than half of the participants perceived their weight accurately (63.6, 53.8, 66.2%, respectively). For overweight and obese adults, older age, male, rural residence, lower level of education, lower level of income, absence of hypertension, presence of diabetes and absence of dyslipidemia positively associated with weight underestimation, and awareness of having hypertension and dyslipidemia were negatively associated with weight underestimation (Adjusted *OR*(*95%CI*) were 0.70(0.61~ 0.79) and 0.71(0.62~ 0.80), respectively). Awareness of having hypertension and dyslipidemia were significantly associated with weight management (Adjusted *OR* (*95%CI*) were 1.42(1.25~ 1.62) and 1.53(1.36~ 1.72), respectively). There was no significant association between awareness of diabetes and weight underestimation(*P* > 0.05) or weight management(*P* > 0.05).

**Conclusions:**

More than half of the participants perceived their weight accurately. For overweight/obese population, awareness of having hypertension and dyslipidemia could improve weight perception and weight management, whereas awareness of having diabetes might not.

## Background

Obesity is an important public health issue worldwide. The prevalence of overweight and obesity has increased since 1980 in developed and developing countries [1]. Obesity has been confirmed to be a risk factor to a variety of diseases such as type 2 diabetes, dyslipidemia, hypertension, coronary heart disease, stroke, and cancer [2–6]. About 3.4 million deaths, 3.9% of years of life lost, and 3.8% of disability-adjusted life years are attributable to overweight and obesity globally in 2010 [7]. In China, the prevalence of overweight and obesity has increased from 12.8 and 3.3% to 17.6% and 5.6% respectively from 1992 to 2002 [8].

It is essential to control body weight within the normal range. The premise of weight management is to perceive self-weight accurately. Previous findings suggest that weight misperception, defined as the difference between actual weight and perceived weight among overweight and obese adults, has an impact on weight-loss attitude and behavior such as physical activity [9]. Misperception of weight could be varied across age, sex, ethnicity, education level and socio-economic status [10–12]. Moreover, weight perception could be affected by one’s comorbidity like diabetes, given the association between overweight and obesity and chronic diseases [13].

Most of the studies on weight misperception have been conducted in developed countries and a few developing countries such as India [14] and Pakistan [15]. Studies in Chinese population are still limited. Since weight perception is a conception affected by culture, region and ethnicity, it is of great need to study weight misperception in Chinese population. In this study, we aimed to describe weight misperception for Chinese adults, to identify factors that may help to explain weight underestimation for overweight and obese individuals, and to investigate whether awareness of having certain chronic diseases could improve weight underestimation and weight management.

## Methods

### Design and participants

This study used data obtained from the 2011 Beijing Non-communicable disease and risk factors Surveillance(BJNCDRS), a cross-sectional survey conducted in 16 districts of Beijing. BJNCDRS sampled Beijing residents aged from 18 to 79 years. Probability-Proportional-to-Size (PPS) and systematic sampling methods were used for the employed population, while PPS and simple random sampling approaches for unemployed and retired population. Details about this survey have been published previously [16].

### Data collection and body weight perception assessment

Demographic characteristics, cigarette smoking, alcohol drinking, dietary intake, physical activity, and disease status were collected via in-person survey interview. Weight, height, waist circumstance and blood pressure were measured individually through physical examination. Fasting peripheral blood samples were obtained and assayed for glucose, total cholesterol, low-density lipoprotein cholesterol(LDL-c), high-density lipoprotein(HDL-c) and triglyceride. More details about BJNCDRS can be found in Reports of the 2011 Beijing Non-communicable disease and risk factors Surveillance [17]. A total of 19,932 participants with measures of weight and height were included in the analysis.

BMI was calculated as weight (in kg) divided by squared height (in m). Individuals were classified as underweight, normal weight, or overweight/obese in the basis of BMI (BMI < 18.5 is underweight; 18.5 ≤ BMI ≤ 24 is normal weight; and BMI > 24 is overweight/obese [18]). Self-perception of weight was obtained by asking “How do you describe your weight?” Response options included “very underweight”, “slightly underweight”, “around normal weight”, “slightly overweight”, “very overweight” and “never considered”. For analysis purposes, these six response options were collapsed into the following categories: underweight (slightly underweight and very underweight), normal weight (around normal weight), overweight (slightly overweight and very overweight) and never considered. According to measured BMI and self-perception, estimation of weight perception was classified into four categories: accurate (BMI and self-perception were in the same level), underestimated (level of self-perception was lower than level of BMI), overestimated (level of self-perception was higher than level of BMI), and N/A (response of the self-perception question was “never considered”). Weight management was measured by asking “Are you taking any actions to control your body weight?”. Response options included “taking some actions to lose weight”, “taking some actions to gain weight”, and “taking no actions”. The participants who chose “taking some actions to lose weight” were defined as weight management groups.

Awareness of having hypertension, diabetes and dyslipidemia were defined as whether patients had realized their relevant morbidities, respectively. Hypertension patients included self-reported individuals taking medicine in the past 2 weeks, and participants whose systolic blood pressure (SBP) ≥140 mmHg and/or diastolic blood pressure (DBP) ≥90 mmHg at the physical examination. Diabetes patients included self-reported diabetes cases, and participants whose fasting glucose ≥7.0 mmol/L. Dyslipidemia patients included self-reported cases, and participants with at least one of the following criteria: total cholesterol ≥240 mg/dl; LDL-c ≥ 160 mg/dl; HDL-c < 40 mg/dl; triglyceride ≥200 mg/dl.

Demographic characteristics included age, sex, residential region, education level and income level, which were also potential confounders.

### Statistical analysis

Descriptive analysis was used to summarize the basic characteristics of the study participants. The variance analysis was used for continuous variables and the chi-square analysis was used for categorical variables. For overweight and obese participants, multiple logistic regression was used to investigate factors associated with weight underestimation, the associations of weight underestimation with hypertension, diabetes and dyslipidemia awareness, and the associations of weight management with weight underestimation and with disease awareness. Odds ratios (*OR*) with 95% confidential intervals (*CI*) were reported. All analyses were performed using the IBM SPSS Statistics version 19.

## Results

### Characteristics of study population

The total number of participants included in the final analysis was 19,932, out of which 459 (2.3%) were underweight, 7392 (37.1%) were normal weight and 12,081 (60.6%) were overweight or obese. The overall mean age of the population was 44.3(SD13.27) years. There were 9033(45.3%) males and 10,899(54.7%) females. Of all, 72.1% (*n* = 14,337) of the participants were urban residents, 53.4% (*n* = 7048) reported college or higher education level as their highest level of education, and 88.6% (*n* = 16,651) earned 1000~ 4999 RMB per month. Seven thousand four hundred eighty-six participants (37.6%) were hypertension patients, 2029 participants (10.2%) were diabetes patients, and 10,233 participants (51.3%) were dyslipidemia patients. Demographic characteristics and morbidities of participants across BMI categories are summarized in Table 1.

**Table 1:** Demographic characteristics and Morbidities of study participants across BMI categories

Characteristics		BMI			*P*†
	Total (*n* = 19,932)	Underweight (*n* = 459)	Normal weight (*n* = 7392)	Overweight/obese (*n* = 12,081)	
Age(year), mean(SD)	44.3(13.27)	34.5(14.49)	41.3(13.48)	46.5(12.56)	< 0.001
Gender, n(%)					< 0.001
Male	9033 (45.3)	129(28.1)	2645(35.8)	6259(51.8)	
Female	10,899 (54.7)	330(71.9)	4747(64.2)	5822(48.2)	
Region, n(%)^a^					0.695
Urban	14,337 (72.1)	325(71.1)	5296(71.8)	8716(72.3)	
Rural	5560 (27.9)	132(28.9)	2082(28.2)	3346(27.7)	
Education^a^, n(%)					< 0.001
Middle school or less	6797 (34.1)	106(23.1)	2068(28.0)	4623(38.3)	
High school	6073 (30.5)	117(25.5)	2206(29.9)	3750(31.1)	
Some college or more	7048 (35.4)	236(51.4)	3113(42.1)	3699(30.6)	
Income(RMB per month)^a^, n(%)					< 0.001
< 1000	1398(7.4)	20(4.7)	350(5.0)	1028(9.0)	
1000~ 4999	16,651 (88.6)	399(92.8)	6369(91.2)	9883(86.8)	
≥ 5000	755 (4.0)	11(2.6)	267(3.8)	477(4.2)	
Hypertension, n(%)					< 0.001
No	12,446(62.4)	421(91.7)	5868(79.4)	6157(51.0)	
Yes	7486(37.6)	38(8.3)	1524(20.6)	5924(49.0)	
Diabetes, n(%)					< 0.001
No	17,903(89.8)	444(96.7)	6979(94.4)	10,480(86.7)	
Yes	2029(10.2)	15(3.3)	413(5.6)	1601(13.3)	
Dyslipidemia, n(%)					< 0.001
No	9699(48.7)	375(81.7)	4835(65.4)	4489(37.2)	
Yes	10,233(51.3)	84(18.3)	2557(34.6)	7592(62.8)	

†*P* < 0.05 indicate that there were differences in the overall distribution of the three groups

^a^There were some missing values in these variables

### Weight misperception

**Fig. 1:**
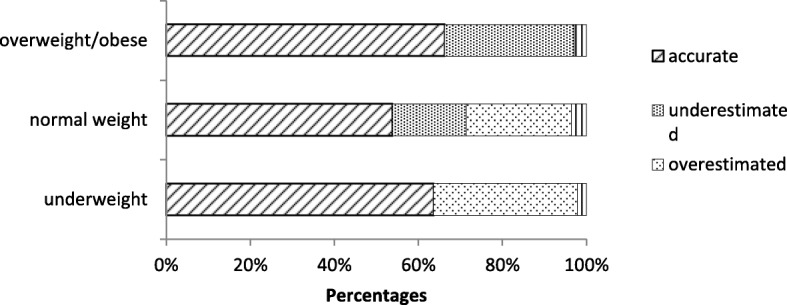
Weight perception by BMI categories

Figure 1 presents the percentage of accuracy weight perception by measured BMI categories. In the underweight, normal weight, and overweight/obese categories, more than half of the participants perceived their weight accurately (63.6% (*n* = 292), 53.8% (*n* = 3979), 66.2% (*n* = 7993), respectively). The proportion of underestimation in the overweight/obese group (30.9% (*n* = 3734)) was higher than the proportion in the normal weight group (17.5% (*n* = 1294)). The proportion of overestimation in the underweight group (34.2% (*n* = 157)) was higher than the proportion in the normal weight group (25.1% (*n* = 1852)).

Factors associated with weight underestimation for overweight and obese participants are shown in Table 2. From the logistic regression, older age, male, rural residence, lower level of education, lower level of income, absence of hypertension, presence of diabetes and absence of dyslipidemia were significantly associated with underestimation of weight.

**Table 2:** Factors associated with weight underestimation among overweight and obese participants

Factors	Weight perception, n(%)		*OR(95%CI)*	*P*
	Accurate	Underestimated		
Age, mean(SD)	44.5(11.94)	50.6(12.81)	1.04(1.04~ 1.05)	< 0.001
Gender				< 0.001
Male	3817(47.8)	2241(60.0)	1	
Female	4176(52.2)	1493(40.0)	0.40(0.36~ 0.43)	
Region				< 0.001
Urban	5993(75.0)	2509(67.4)	1	
Rural	1993(25.0)	1213(32.6)	1.36(1.22~ 1.53)	
Education				< 0.001
Middle school or less	2551(31.9)	1887(50.5)	1	
High school	2523(31.6)	1116(29.9)	0.76(0.68~ 0.85)	< 0.001
Some college or more	2912(36.5)	730(19.6)	0.52(0.46~ 0.59)	< 0.001
Income				< 0.001
< 1000	501(6.6)	486(13.8)	1	
1000~ 4999	6683(88.7)	2918(82.9)	0.69(0.59~ 0.80)	< 0.001
≥ 5000	354(4.7)	118(3.4)	0.64(0.49~ 0.84)	0.001
Hypertension				< 0.001
No	4190(52.4)	1778(47.6)	1	
Yes	3803(47.6)	1956(52.4)	0.71(0.64~ 0.77)	
Diabetes				0.001
No	7083(88.6)	3095(82.9)	1	
Yes	910(11.4)	639(17.1)	1.23(1.09~ 1.40)	
Dyslipidemia				< 0.001
No	2766(34.8)	1496(40.4)	1	
Yes	5171(65.2)	2208(59.6)	0.63(0.58~ 0.70)	

Table 3 displays association of weight underestimation with disease awareness for hypertension, diabetes and dyslipidemia patients in the overweight and obese participants. Awareness of having hypertension and dyslipidemia were negatively associated with underestimation of weight perception (*OR* (*95%CI*) =0.90(0.80~ 1.00), 0.78(0.70~ 0.87), respectively), whereas no significant association was found between awareness of having diabetes and weight underestimation. After adjusted for age, sex, region, education and income, the results remained similar (Adjusted *OR*(*95%CI*) for awareness of having hypertension and dyslipidemia were 0.70(0.61~ 0.79) and 0.71(0.62~ 0.80), respectively).

**Table 3:** Association of weight underestimation with the awareness of disease among hypertension, diabetes and dyslipidemia patients in overweight and obese participants

Awareness		Weight perception, n(%)		*OR(95%CI)*	*P*	OR(95%CI)*	P*
		Accurate	Underestimated				
Hypertension	no	1827(48.1)	994(50.8)	1		1	
	yes	1975(51.9)	962(49.2)	0.90(0.80~ 1.00)	0.047	0.70(0.61~ 0.79)	< 0.001
Diabetes	no	359(39.5)	233(36.5)	1			
	yes	551(60.5)	406(63.5)	1.14(0.92~ 1.40)	0.234	1.05(0.83~ 1.33)	0.663
Dyslipidemia	no	3478(67.3)	1601(72.5)	1			
	yes	1693(32.7)	607(27.5)	0.78(0.70~ 0.87)	< 0.001	0.71(0.62~ 0.80)	< 0.001

*Adjusted for age, gender, region, education and income

### Weight management

The associations of weight management with weight underestimation and with awareness of having hypertension, diabetes and dyslipidemia for overweight and obese participants are presented in Table 4. Compared with the weight management group, the proportion of weight underestimation was higher in the non-management group (12.0% v.s. 40.0%, Adjusted *OR* (*95%CI*) =0.25(0.22~ 0.28)). Awareness of having hypertension and dyslipidemia were significantly associated with weight management (Adjusted *OR* (*95%CI*) 1.42(1.25~ 1.62) and 1.53(1.36~ 1.72), respectively), whereas awareness of having diabetes was not.

**Table 4:** Association of weight management with weight underestimation and the awareness of hypertension, diabetes and dyslipidemia among overweight and obese participants

Factors	Weight management, n(%)			*OR(95%CI)*	*P*	OR(95%CI)*	P*
	No		Yes				
Weight underestimation	no	4990(60.0)	3003(88.0)	1		1	
	yes	3324(40.0)	410(12.0)	0.21(0.18~ 0.23)	< 0.001	0.25(0.22~ 0.28)	< 0.001
Hypertension awareness	no	2171(50.0)	748(47.4)	1		1	
	yes	2172(50.0)	831(52.6)	1.11(0.99~ 1.25)	0.075	1.42(1.25~ 1.62)	< 0.001
Diabetes awareness	no	473(39.1)	151(38.7)	1		1	
	yes	738(60.9)	239(61.3)	1.01(0.80~ 1.28)	0.904	1.07(0.83~ 1.38)	0.630
Dyslipidemia awareness	no	3827(71.3)	1431(64.3)	1		1	
	yes	1538(28.7)	796(35.7)	1.38(1.25~ 1.54)	< 0.001	1.53(1.36~ 1.72)	< 0.001

*Adjusted for age, gender, region, education and income

## Discussion

Although obesity has been recognized as a risk factor of multiple diseases, the accuracy of weight perception in Chinese population is still unclear. In this study, we described the weight perception status for adults in Beijing, and identified that age, sex, education level, income level and comorbidities are associated with weight underestimation. Moreover, our results showed that awareness of having hypertension and dyslipidemia were negatively associated with weight underestimation and positively associated with weight management.

For the 19,932 participants, more than half (60.6%) were overweight and obese according to their measured BMI. The prevalence rate of overweight and obesity for Chinese adults was 42.6% in 2010, and the rate of overweight and obesity in urban population was higher than those in rural population. The rate showed a gradually decreasing trend from eastern, central to western regions [16]. Although there was no direct comparison, Beijing, as the capital and an economically developed region, had a high overweight and obesity rate, which was in line with the relatively high prevalence of overweight and obesity in China. For the overweight and obese participants in Beijing, 66.2% perceived their weight status accurately. Similar proportions have been reported before. In a study of Caucasians, Latinos, Filipinos and Koreans, two thirds overweight and obese participants perceived their weight status correctly [19]. The proportion of weight misperception was reported to be 35.9% for overweight and obese Mexican American men according to the result of the 1999–2006 National Health and Nutrition Examination Survey [20]. In a study of Pakistani, a higher proportion (50%) of weight misperception was reported. This could be explained by the desired attitude for overweight or obesity in South Asian culture [15]. Nevertheless, overweight and obese individuals are more likely to underestimate their weight. A similar finding was reported that the proportion of individuals having underestimated perception was larger in overweight group than in healthy-weight group by a US study [12]. This phenomenon is noteworthy, for that these overweight and obese individuals who are at high risk for chronic diseases do not care about their actual weight status or not realize the hazard of obesity. It is suggested that people with normal or under-normal weight are more likely to care about their body weight, given these people had a higher proportion of overestimating their body weight in our study. By contrast, overweight and obese individuals are more likely to neglect the body weight and its relative risk.

The differences in weight misperception by age, sex, education level and socio-economic status have been reported in previous studies [10–12, 21–23]. We also found that older participants, males, participants with lower level of education and participants with lower level of income were more likely to underestimate their weight. These consistent results implicate that there is a similar distribution of the attitude on overweight and obesity across age, sex, education level and socio-economic status in different countries and regions. One example of such consistency is that males are more likely to underestimate their weight across countries and regions. The possible explanation to the greater underestimation in males is that social and cultural factors, and the social concept of “lean as beauty” have a greater impact on females, as a result, females are more concerned about their own weight compared with males. Moreover, we found that rural residents had a higher odds of weight underestimation compared with urban residents. A similar finding has also been reported in a study conducted in Guangdong Province, China [24]. And in the PERU MIGRANT study, rural residents had the lowest Kappa coefficient between BMI and self-reported weight compared with urban residents and rural-to-urban migrants [25]. Residential region could be an indicator of social and cultural factors.

In addition to the associations of weight underestimation with demographic characteristics, we investigated the associations with the presence and awareness of hypertension, diabetes and dyslipidemia. Our results indicate that participants with hypertension or dyslipidemia are more likely to realize their actual weight, whereas participants with diabetes are more likely to underestimate their weight. Overweight and obesity are risk factors of hypertension and dyslipidemia, and weight loss has beneficial effects on prevention and control of hypertension and dyslipidemia [26–30]. From our subsequent analyses, awareness of having hypertension and dyslipidemia showed higher odds of accurate weight perception and weight management. These results suggest that participants could realize their lifetime risks for hypertension and dyslipidemia resulting from overweight and obesity. Paradoxically, no association between awareness of having diabetes and weight underestimation or weight management was observed, though obesity is also a serious risk factor for diabetes [31]. Similar results have also been reported by previous studies [32, 33]. One possible explanation to this paradox is that although benefits of lifestyle modification and weight loss on diabetes treatment have been verified in previous studies [34, 35], many participants might be influenced by the ancient common sense in China that diabetes patients would become thinner. Presence of diabetes mellitus was not associated with increased awareness of actual weight status in a Pakistan study [15] and Mogre et al. reported that participants being hyperglycemic were not significantly associated to underestimation of weight [32]. These results implicate that risk for diabetes caused by obesity had not been paid much attention to. Furthermore, public awareness of different diseases and their risk factors is insufficient, and this would make prevention and treatment efforts more challenging.

This is a relatively large study in Chinese population on weight misperception. The standard questionnaire and physical examination is implemented for each participant. However, there are still a few limitations in this study. First, oral glucose tolerance test (OGTT) has not been used for diabetes diagnosis so that there might be misclassification of diabetes patients. Second, although weight perception might be different between Han people and minorities, most of participants enrolled in our study are Han people and the sample size is insufficient for an analysis for minorities. Third, we investigated whether control of blood pressure and glucose for hypertension and diabetes patients associated with weight perception and weight management, but there was no significant association (data not shown). The reasons might be the number of participants that could be included in analyses was relatively small, or it is difficult to identify the relationship between control of blood pressure and glucose and weight perception or management, since control of blood pressure and glucose may be influenced by many kinds of factors.

## Conclusions

More than half Beijing adults perceive their weight accurately. For the overweight and obese population, the hazards from obesity for hypertension and dyslipidemia have been realized, while hazard from obesity for diabetes has not. It should be useful for accurate weight perception and weight management by improving awareness of hypertension and dyslipidemia. Further studies are needed to investigate the association of weight perception with presence and awareness of diabetes. Patients should be provided information of health risk associated obesity, and the risk of obesity on diabetes should be paid attention to seriously.

## Abbreviations

BJNCDRS: Beijing Non-communicable disease and risk factors Surveillance
*CI*: Confidential intervals
HDL-c: High-density lipoprotein
LDL-c: Low-density lipoprotein cholesterol
*OR*: Odds ratios
